# Prognostic value of Amyloid PET in Real-World Amnestic MCI: Influence of reader experience and interpretation method

**DOI:** 10.1007/s00259-026-08159-9

**Published:** 2026-08-28

**Authors:** Beatriz Echeveste, Edgar Fernando Guillén, Elena Prieto, Katharina C. E. Hirschmüller, Mario Riverol, Javier Arbizu

**Affiliations:** 1 Neurology Department, University of Navarra Clinic, Pamplona, Spain; 2 Nuclear Medicine Department, University of Navarra Clinic, Pamplona, Spain; 3 Medical Physics. Nuclear Medicine Department, University of Navarra Clinic, Pamplona, Spain

**Keywords:** Alzheimer’s disease, Amnestic mild cognitive impairment, Amyloid PET, Visual interpretation, SUVR, Reader expertise, Inter-observer agreement

## Abstract

**Purpose:**

This study assessed the prognostic value and inter-/intra-observer reliability of three amyloid PET interpretation methods—conventional visual reading, assisted visual interpretation and standardized uptake value ratio (SUVR)-based quantification—for predicting conversion to Alzheimer’s disease (AD).

**Methods:**

This retrospective longitudinal study included 145 patients with amnestic mild cognitive impairment who underwent amyloid PET and were followed for up to 8 years at a single memory clinic. Two certified nuclear medicine physicians performed structured visual reads and assisted visual interpretations. Semiquantitative SUVR thresholds validated in prior studies were applied as a third method. Inter- and intra-observer agreement was assessed using Cohen’s kappa (κ). Cox regression models estimated the association of conversion to AD in PET-positive versus PET-negative patients for each method, adjusting for age, sex and education.

**Results:**

Inter-observer agreement was excellent for structured visual reading (κ = 0.877) and assisted visual interpretation (κ = 0.904). Intra-observer reliability was also high (κ = 0.891 for Reader 1; κ = 0.859 for Reader 2). Consensus agreement for the assisted method reached κ = 0.936. Amyloid PET positivity was strongly associated with progression to AD across all methods. Hazard ratios were highest for assisted visual interpretation (HR = 3.23, 95% CI 1.94–5.38, *p* < 0.0001), followed by SUVR quantification (HR = 2.19, 95% CI 1.55–3.10, *p* < 0.0001) and structured visual reading (HR = 1.83, 95% CI 1.10–3.06, *p* = 0.02).

**Conclusion:**

Amyloid PET positivity consistently predicted conversion to AD regardless of interpretation method. Assisted visual and semiquantitative approaches showed numerically higher prognostic discrimination, while expert structured visual reading remained clinically reliable.

## Introduction

Amnestic mild cognitive impairment (aMCI) is a prodromal phase of Alzheimer´s disease (AD) associated with a high risk of progression to dementia [1]. Amyloid positron emission tomography (PET) identifies individuals with underlying β-amyloid pathology, who are more likely to experience clinical decline. However, scan interpretation is often challenging in borderline cases or with low-to-intermediate tracer retention [2]. Quantitative support for amyloid PET analysis includes cortical region-based z-score mapping and comparisons with age-matched normal databases, and standardised uptake value ratio (SUVR) measurements among others [3]. These approaches are increasingly available in clinical practice and are recommended in the updated Appropriate Use Criteria as complementary tools to visual interpretation in selected clinical scenarios [4]. Nevertheless, whether these approaches improve reproducibility or prognostic performance beyond expert visual interpretation remains uncertain, particularly in early stages such as amnestic mild cognitive impairment. Recently a large real-world study found a high concordance between local visual reads and central quantification of clinical amyloid-PET scans [5].

Although visual reading is the current clinical standard, it is subject to inter-observer and intra-observer variability, potentially limiting prognostic reliability and reproducibility [6]. Semi-quantitative tools, including SUVR and z-score maps, aim to reduce subjectivity by providing standardised, reproducible metrics anchored to predefined thresholds [2].

Previous studies suggest that adding semi-quantitative overlays or SUVR cutoffs improves reader agreement and sensitivity for detecting amyloid positivity [7]. However, few studies have directly compared visual-only interpretation, visual assisted interpretation and SUVR-based quantification in longitudinal aMCI cohorts with clinical follow-up [8].

In this real-world clinical study, we evaluated how three interpretation strategies (structured visual reading, assisted visual interpretation and SUVR-based quantification) differ in terms of agreement, reproducibility and prediction of progression from aMCI to AD dementia. We aimed to assess intra-observer and inter-observer variability for visual and assisted visual methods and compared the prognostic performance of all three methods for predicting conversion from aMCI to AD dementia during longitudinal follow-up. Integrating semi-quantitative tools into clinical workflows may improve standardisation of amyloid PET interpretation and enhance early risk stratification in patients with aMCI.

## Methods

### Study design and participants

We conducted a retrospective longitudinal study with follow-up of up to 8 years in routine clinical practice. Between October 2013 and March 2021, we included patients with a clinical diagnosis of aMCI [9] evaluated at the Memory Unit of Clínica Universidad de Navarra who underwent amyloid PET as part of their diagnostic workup.

All participants were assessed by a neurologist specialised in cognitive disorders. The baseline evaluation included a detailed medical history, an informant-based interview, a neurological examination and standard laboratory testing (complete blood count, biochemistry, vitamin B12, serum folate, glucose, lipid profile, syphilis serology and thyroid function tests). All patients also underwent brain magnetic resonance imaging (MRI) and a comprehensive neuropsychological assessment.

Exclusion criteria were age > 85 years, current depression or dysthymia, abnormal laboratory findings, significant medical conditions or substance abuse affecting cognition and alternative causes of focal or diffuse brain injury on MRI, including lacunar infarcts or extensive cerebrovascular disease.

Patients were followed longitudinally to determine progression to AD dementia [10]. All participants provided written informed consent for the use of their clinical data in research. This study was reviewed and approved by the Ethics Research Committee of the University of Navarra, with no ethical concerns reported.

### Amyloid-PET imaging

#### Patient preparation, radiotracer administration and image acquisition

No specific patient preparation or medication adjustments were required before amyloid PET. Peripheral venous access was established in the upper limb and the radiotracer was administered according to compound-specific technical guidelines and standard procedural recommendations for amyloid imaging. Three ^18^F-labelled radiotracers were used: Florbetapir (*n* = 79), Florbetaben (*n* = 20) and Flutemetamol (*n* = 46).

PET acquisitions were performed at tracer-specific uptake times following intravenous injection: 50 min after 370 MBq of [^18^F]-Florbetapir, 90 min after 240–360 MBq of [^18^F]-Florbetaben and 90 min after 185 MBq of [^18^F]-Flutemetamol. Emission PET acquisition lasted 20 min for all three radiotracers. All scans were obtained in 3D mode on a Biograph mCT PET/CT scanner (Siemens Healthineers). Images were reconstructed with an iterative algorithm incorporating time-of-flight and point spread function modelling, using 3 iterations and 21 subsets, with a 2-mm Gaussian filter and a zoom factor of 2. Although motion correction was not applied, all PET examinations were visually assessed immediately after acquisition to ensure that the images were of an adequate quality and that there were no clinically relevant motion artefacts present.

#### Visual analysis

Visual interpretation was performed using the syngo.via^®^ platform (Siemens Healthineers) in accordance with the manufacturer’s guidelines for each radiotracer. Two nuclear medicine physicians independently reviewed all amyloid PET examinations: an experienced reader (JA, Reader 1) with over 30 years of experience in nuclear medicine and a reader with 5 years of experience (FG, Reader 2). Both were certified to visually interpret amyloid PET scans using Florbetapir, Florbetaben and Flutemetamol. All images were evaluated using tracer-specific interpretation criteria and both readers were blinded to clinical information.

**Fig. 1:**
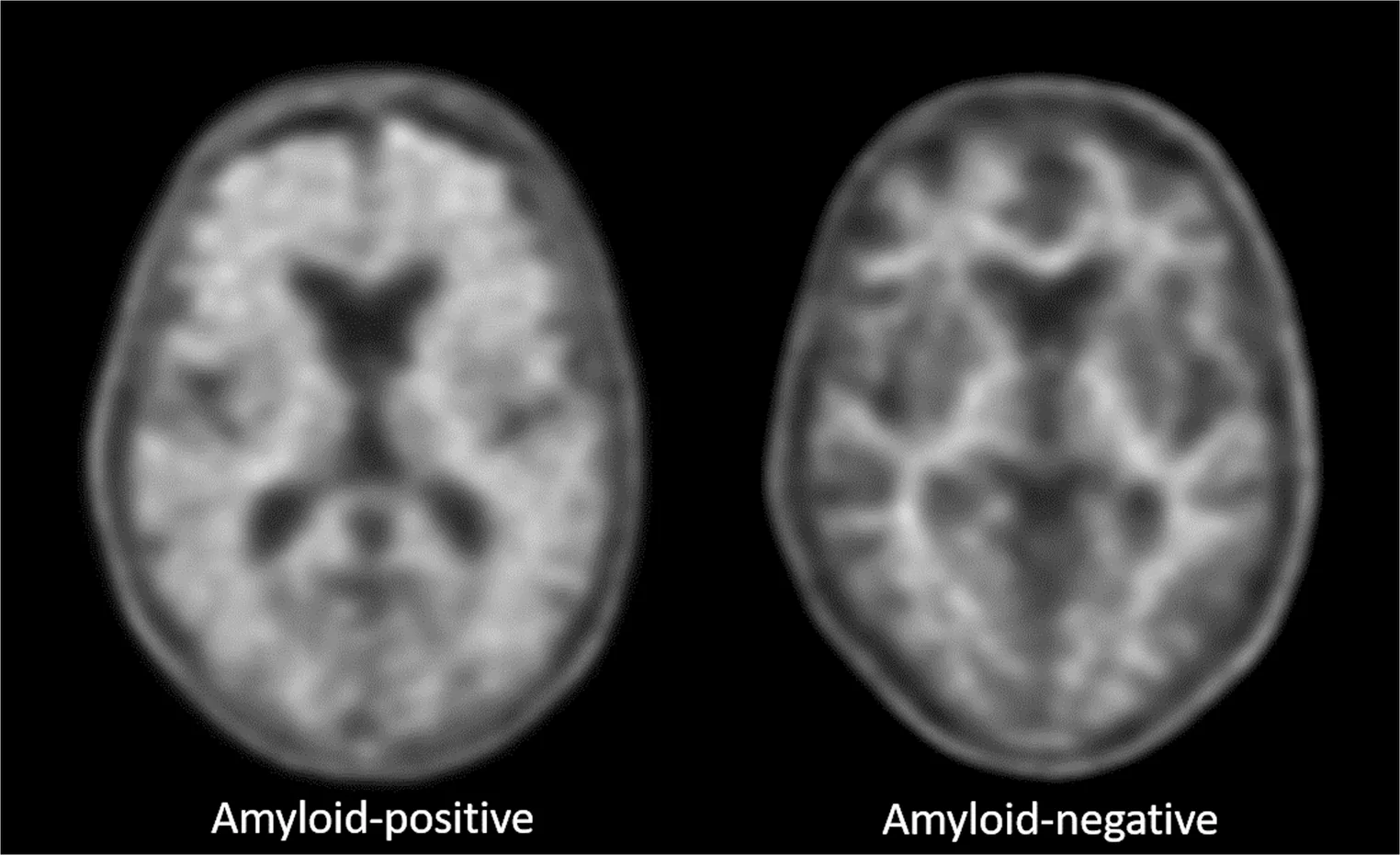
Representative florbetapir PET images interpreted using the standard visual assessment method. The left panel shows an amyloid-positive scan, characterized by increased cortical tracer retention consistent with significant β-amyloid deposition. The right panel depicts an amyloid-negative PET, demonstrating preserved grey–white matter contrast and absence of pathological cortical uptake

Scans were classified as amyloid-positive (A+) or amyloid-negative (A−) according to each radiotracer’s technical data sheet (Fig. 1). Discrepancies between the two readers were resolved by consensus for the posterior Cox analysis.

#### Assisted visual analysis

**Fig. 2:**
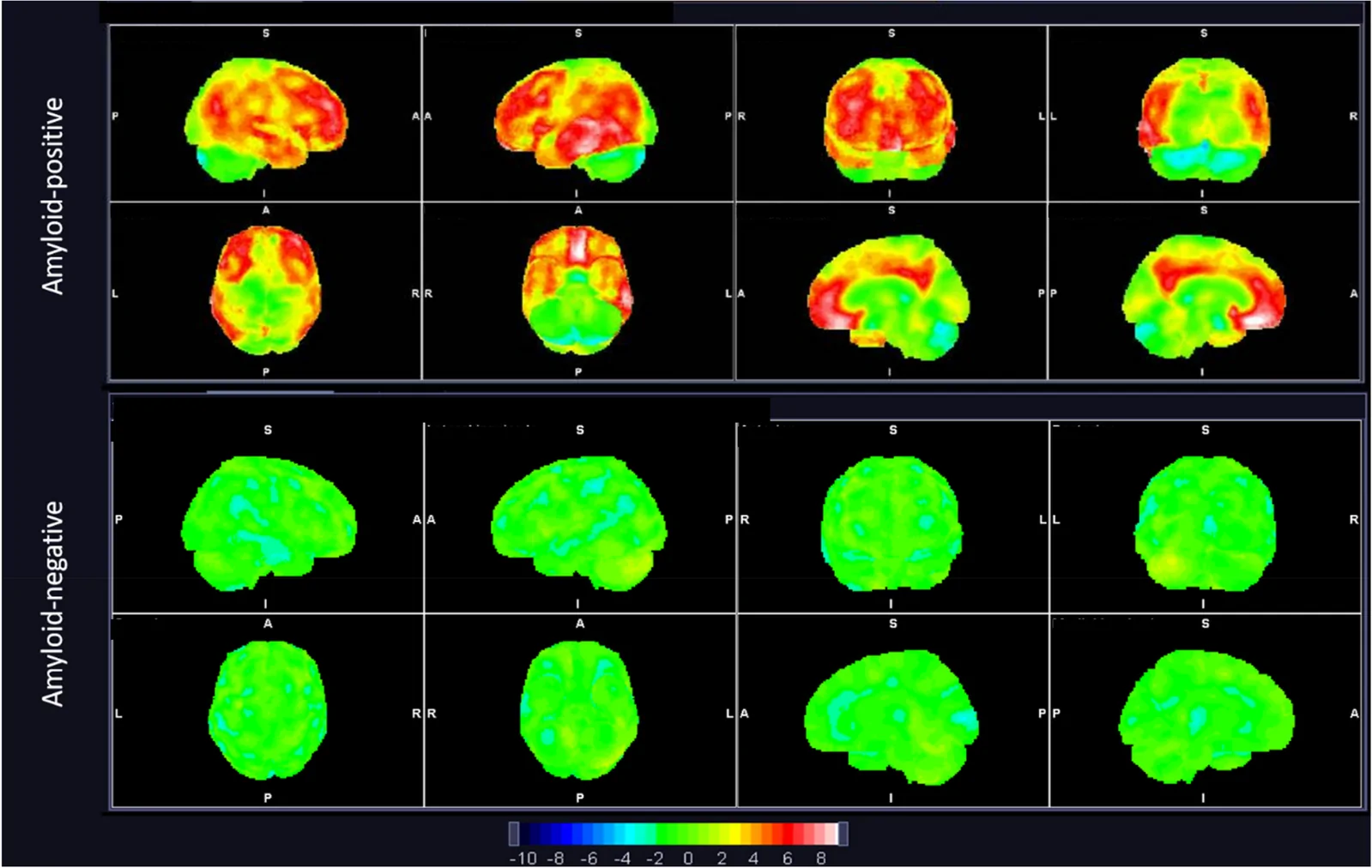
Representative examples of assisted amyloid PET interpretation. Statistical projection maps illustrating an amyloid-positive case (top), with diffuse cortical tracer retention, and an amyloid-negative case (bottom), without abnormal cortical uptake. Values are displayed relative to the normal database in standard deviation units

In a second step, both readers re-evaluated the same PET images using z-score maps and statistical overlays generated by the syngo.via^®^ MI Neuro Cortical Analysis software (Siemens Healthineers) [11]. These maps are based on tracer-specific normative databases integrated into the software and provide voxel-wise statistical comparisons with normal reference populations. The implementation follows the manufacturer’s recommendations for each radiotracer. This approach guided visual interpretation through voxel-wise statistical comparisons, highlighting regions with abnormally increased tracer uptake relative to normal controls (Fig. 2).

The protocol consisted of readers first performing an independent visual interpretation of each anonymized amyloid PET scan. They then re-evaluated the same examination based on quantitative information (z-score maps and SUVR values), reflecting the intended clinical workflow in which quantitative tools objectively complement the initial visual assessment. Accordingly, no formal washout period was introduced between the two interpretation strategies.

#### Semi-quantitative Analysis

SUVRs were calculated using the default tracer-specific composite cortical regions implemented in the syngo.via^®^ MI Neuro Cortical Analysis software. The composite regions comprised the anterior cingulate, frontal, parietal, posterior cingulate and temporal cortices, together with the precuneus for florbetapir and flutemetamol, and the occipital cortex for florbetaben. SUVRs were normalized using the manufacturer-recommended tracer-specific reference regions (cerebellum for florbetapir, whole cerebellum for florbetaben, and pons for flutemetamol). Scans were classified as amyloid-positive if SUVR exceeded literature-validated thresholds: >1.17 for Florbetapir [12], > 1.35 for Florbetaben [13] and > 0.60 for Flutemetamol [14]. These cutoff values were established in prior studies and correspond to optimal discrimination between amyloid-positive and amyloid-negative states.

### Criteria for clinical progression

Patients were followed at the Memory Unit of the Neurology Department, Clínica Universidad de Navarra, until January 2022. Follow-up included clinical evaluations by a neurologist specialised in cognitive disorders and repeated neuropsychological assessments. Patients with less than 6 months of clinical follow-up were excluded from the longitudinal analyses.

Clinical progression was defined by fulfilment of the National Institute on Ageing–Alzheimer’s Association (NIA-AA) 2011 criteria for AD dementia [10]. For patients who developed other forms of dementia, a diagnostic re-evaluation was performed to determine whether they met established criteria for alternative neurodegenerative disorders [15–19].

Only patients meeting the criteria for AD dementia were considered to have reached the primary progression endpoint. Patients who had not progressed to Alzheimer’s disease dementia by their last available clinical assessment were treated as censored observations in the Cox proportional hazards models.

### Statistical analyses

Descriptive statistical analyses were performed using Statistical Package for the Social Sciences, version 23, while prospective and correlational statistical analyses were conducted using Software for Statistics And Data Science, version 15. All analyses were adjusted for age, sex and education level, with significance set at *p* < 0.05. Baseline demographic and clinical characteristics were summarised using means and standard deviations for normally distributed continuous variables or medians and interquartile ranges (IQRs) for non-normal variables. Inter- and intra-observer agreement was measured assessed with Cohen’s kappa (κ). To compare prognostic performance among amyloid PET interpretation methods, three Cox proportional hazards models were constructed using visual assessment, assisted visual classification and SUVR-based quantification as independent predictors of clinical progression to AD. Each model was adjusted for sex, age at diagnosis and years of education. Kaplan–Meier curves were constructed to illustrate time to progression to Alzheimer’s disease dementia according to amyloid PET status for each interpretation method. Survival distributions between PET-positive and PET-negative groups were compared using the log-rank test.The amyloid PET-negative group served as the reference category in all Cox proportional hazards models. Hazard ratios therefore represent the relative risk of progression to Alzheimer’s disease associated with amyloid PET positivity. As a secondary exploratory analysis, we examined the studies showing disagreement between conventional visual and visually assisted interpretation across individual readers, this analysis was considered exploratory and descriptive. Given the limited number of discordant cases (*n* = 18), no formal statistical comparisons between interpretation methods were performed. Clinical progression to Alzheimer’s disease dementia during follow-up was used as a pragmatic reference standard, such that a classification was considered correct when an amyloid-positive scan was followed by progression to AD or when an amyloid-negative scan remained clinically stable, and incorrect in the opposite scenarios.

## Results

### Population

Between October 2013 and March 2021, 145 patients with aMCI who underwent amyloid PET imaging were included (Table 1). The mean age at baseline was 71.31 years (95% CI: 58.86–83.78), with 85 females (58.6%) and 60 males (41.4%). The mean Mini-Mental State Examination (MMSE) score at initial evaluation was 26.17 (range: 21–30) and the mean years of formal education was 11.18 (95% CI: 4–19.18).

**Table 1:** Demographic and clinical characteristics of the 145 patients with aMCI included in the study

Variable	Value
No.	145
Age, mean (IQR), y	71.31 years (58.86–83.78)
Sex, No. (%)	
Female	85 (58.6)
Male	60 (41.4)
MMSE, mean (range), score	26.17 (21–30)
Education, mean (IQR), y	11.18 (4–19.18)
Hypertension, (%)	48
Diabetes mellitus, (%)	15.17
Dyslipidemia, (%)	60.68
Active smoking, (%)	8.96
Former smokers (%)	33.10
Antihypertensive treatment (%)	44.13
Benzodiazepines treatment (%)	31.03
Antidepressant treatment (%)	34.38
Family history of AD	
Maternal (%)	2.75
Paternal (%)	9.65

*No* Number, *IQR* Interquartile range, *MMSE* Mini-Mental State Examination, *AD* Alzheimer´s disease

### Patient follow-up and progression to Alzheimer’s disease dementia

**Fig. 3:**
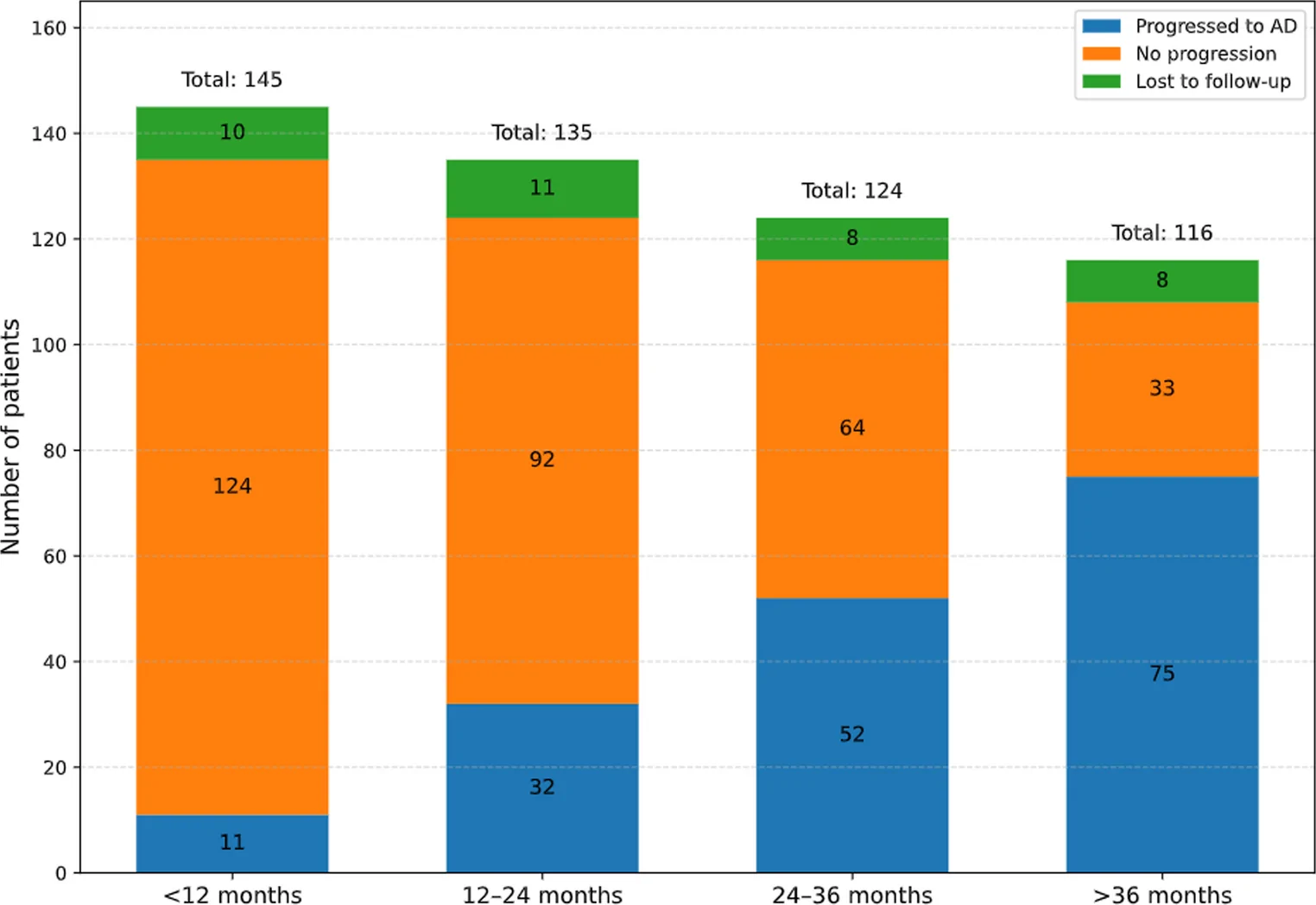
Longitudinal progression to Alzheimer’s disease over 8 years

Clinical progression to AD was tracked at regular follow-up intervals (Fig. 3). Within the first 12 months, 11 patients converted to AD, 134 remained stable and 10 patients were lost to follow-up. Between 12 and 24 months, 32 patients progressed, 103 remained stable and 11 were lost. Between 24 and 36 months, 52 patients progressed to AD, 72 remained stable and 8 were lost. After 36 months of follow-up, a total of 75 patients had converted to AD, 41 remained stable and 8 were lost during this final interval.

Overall, 29 patients were excluded from longitudinal progression analyses due to loss to follow-up, including those who did not attend scheduled clinical assessments and those who developed non-AD dementias. Figure 3 illustrates the timeline of AD progression, stable cases and attrition across follow-up periods.

### Inter- and intra-observer agreement in Amyloid PET interpretation

#### Inter-observer Variability

Using structured visual interpretation for each radiotracer, Reader 1 classified 94 scans as amyloid-positive and 51 as amyloid-negative, whereas Reader 2 classified 96 as positive and 49 as negative. Inter-observer agreement was excellent, with a Cohen’s kappa of 0.877 (95% CI: 0.781–0.951).

With assisted visual interpretation, Reader 1 classified 101 scans as amyloid-positive and 44 as negative, whereas Reader 2 classified 97 as positive and 48 as negative. Although some differences in classification were observed, inter-observer agreement remained excellent, with a Cohen’s kappa of 0.904 (95% CI: 0.815–0.968).

#### Intra-observer Variability

To assess consistency across interpretation approaches, both specialists were compared according to their classifications obtained with structured visual reading and assisted visual interpretation. For Reader 1, agreement between visual and assisted interpretation showed 43 concordant amyloid-negative cases and 93 concordant amyloid-positive cases, with discordance in 9 scans overall, yielding a Cohen’s kappa of 0.860 (95% CI: 0.802–0.957), indicating excellent agreement.

For Reader 2, agreement between visual and assisted interpretation showed 45 concordant amyloid-negative cases and 93 concordant amyloid-positive cases, with discordance in 7 scans overall. Cohen’s kappa was 0.892 (95% CI: 0.760–0.937), also reflecting excellent agreement.

**Fig. 4:**
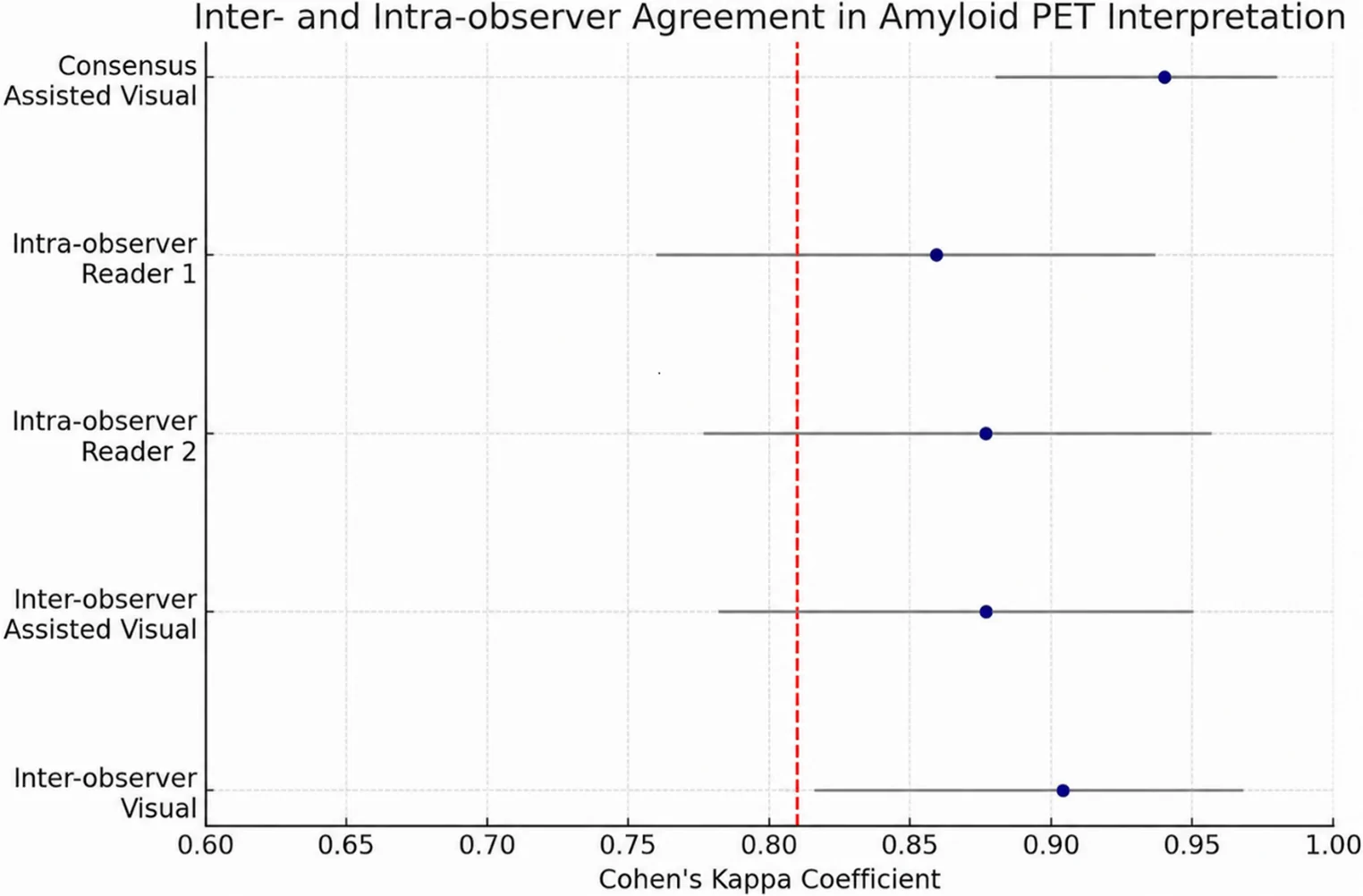
Inter- and intra-observer agreement for visual and assisted visual interpretation of amyloid PET scans, expressed as Cohen’s kappa coefficients with 95% confidence intervals. The red dashed line indicates the threshold for 'excellent agreement (κ > 0.81)

When final consensus ratings were established using the assisted visual method, overall inter-reader agreement reached a Cohen’s kappa of 0.936 (95% CI: 0.862–0.985), indicating near-perfect agreement in the classification of amyloid positivity. These results were used for the Cox analysis. Results are presented in Table 2; Fig. 4.

**Table 2:** Contingency tables showing inter- and intra-observer agreement for amyloid PET interpretation using visual and visually assisted assessment

Inter-observer: Visual reading		
R2 \ R1	**A (-)**	**A (+)**
A (-)	46	3
A (+)	5	91
Inter-observer: Visualy asssited		
R2 \ R1	**A (-)**	**A (+)**
A (-)	43	5
A (+)	1	96
Intra-observer: reader 1		
Assisted\Visual	**A (-)**	**A (+)**
A (-)	43	1
A (+)	8	93
Intra-observer: reader 2		
Assisted\Visual	**A (-)**	**A (+)**
A (-)	45	3
A (+)	4	93

Inter-observer agreement between reader 1 (R1) and reader 2 (R2) is shown for visual reading and visually assisted reading. Intra-observer agreement between visual and visually assisted assessment is shown for reader 1 and reader 2 Classifications are reported as amyloid positive (A+) or negative (A-)

#### Comparative analysis of Amyloid PET classification methods in predicting conversion to Alzheimer’s Disease among PET-Positive patients

**Fig. 5:**
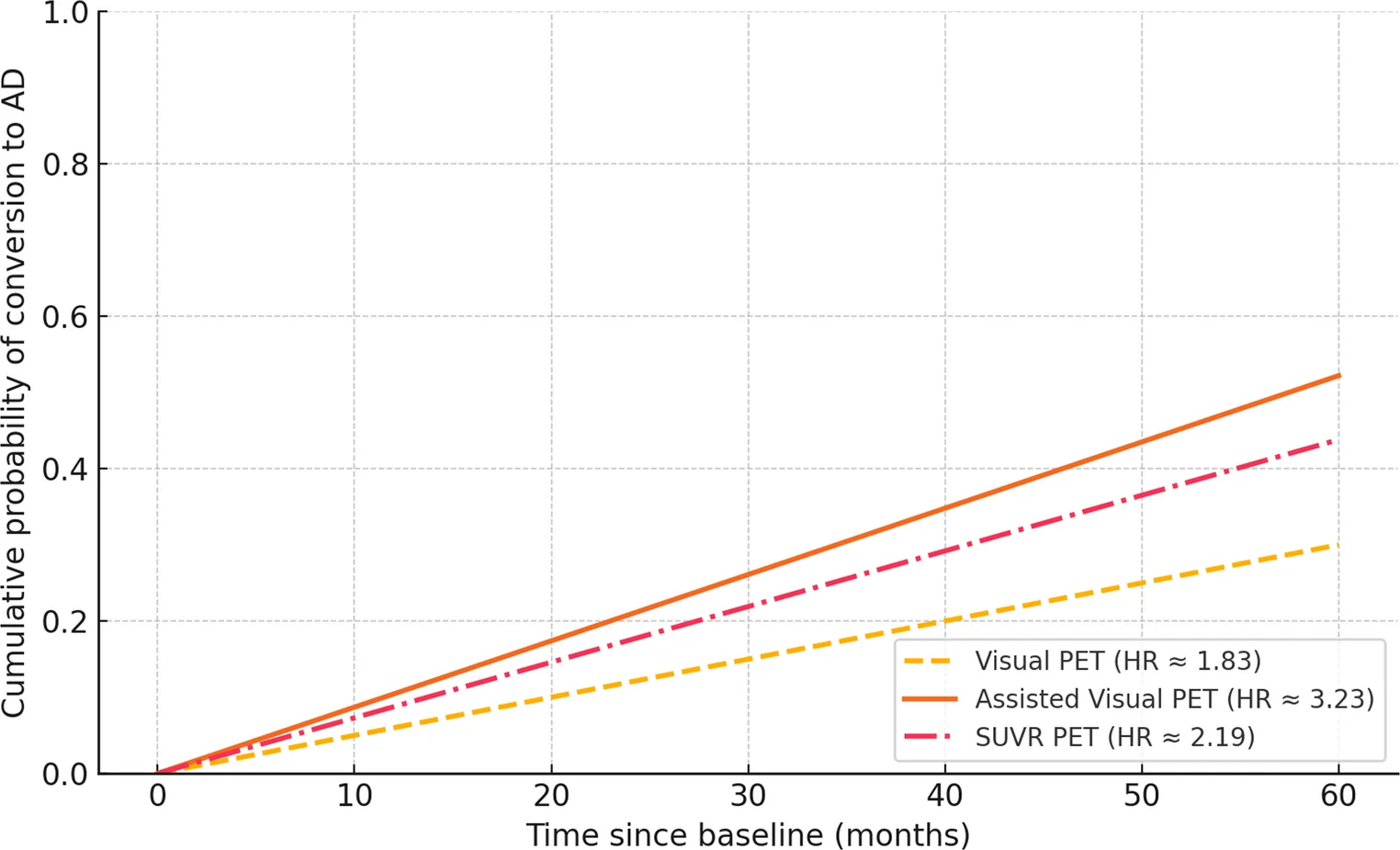
Cumulative probability of conversion to Alzheimer’s disease (AD) among patients with a positive amyloid PET scan, stratified by method of interpretation

In the visual interpretation model, amyloid PET-positive patients exhibited a significantly higher risk of progression to AD than PET-negative patients (HR = 1.83, 95% CI: 1.10–3.06, *p* = 0.020), corresponding to an 83% increase in conversion risk (Fig. 5).

The assisted visual model, which integrates normative z-score overlays to enhance detection accuracy, showed the strongest association. The PET-positive group had a 3.23-fold greater risk of AD conversion than the PET-negative group (HR, 3.23; 95% CI: 1.94–5.38, *p* < 0.001), yielding the highest hazard ratio among the three interpretation methods.

The SUVR-based model also significantly predicted clinical progression. The PET-positive group had a 2.19-fold higher risk of progression than the PET-negative group (HR for PET-negative = 0.456; inverse HR = 2.19, 95% CI: 1.55–3.10, *p* < 0.001). SUVR classification also showed strong prognostic performance.

All three models demonstrated excellent overall fit (omnibus test *p* < 0.001). Among covariates, sex reached statistical significance only in the assisted visual model, with males showing a lower risk of progression than females (HR = 0.546, *p* = 0.020).

**Fig. 6:**
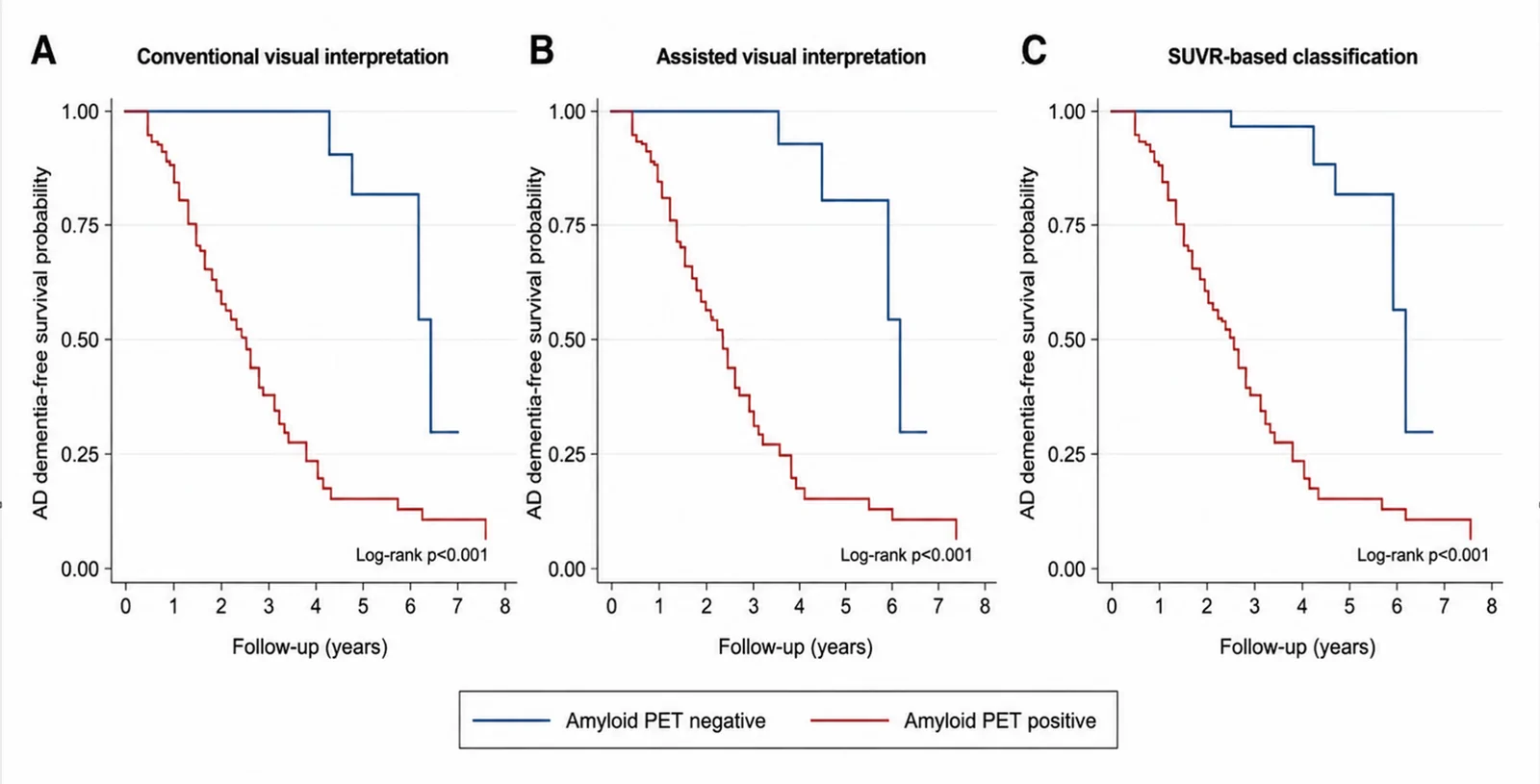
Kaplan–Meier curves for progression to Alzheimer's disease dementia according to amyloid PET status. Kaplan–Meier survival curves are shown for (**A**) conventional visual interpretation, (**B**) assisted visual interpretation, and (**C**) SUVR-based classification. Survival distributions differed significantly between PET-positive and PET-negative groups for conventional visual interpretation (log-rank χ² = 37.76, *p* < 0.001), assisted visual interpretation (log-rank χ² = 35.31, *p* < 0.001), and SUVR-based classification (log-rank χ² = 29.32, *p* < 0.001)

Kaplan–Meier survival analyses confirmed a significantly higher probability of progression to AD dementia among amyloid PET-positive patients for all three interpretation methods (Fig. 6). The separation between PET-positive and PET-negative survival curves was significant for conventional visual interpretation (log-rank χ²=37.76, *p* < 0.001), assisted visual interpretation (χ²=35.31, *p* < 0.001) and SUVR-based classification (χ²=29.32, *p* < 0.001) (Fig. 6).

Overall, amyloid PET positivity was an independent predictor of progression from aMCI to AD across all methods. The assisted visual method produced the highest estimated risk, followed by SUVR quantification and then conventional visual assessment.

#### Inter-observer disagreement

**Fig. 7:**
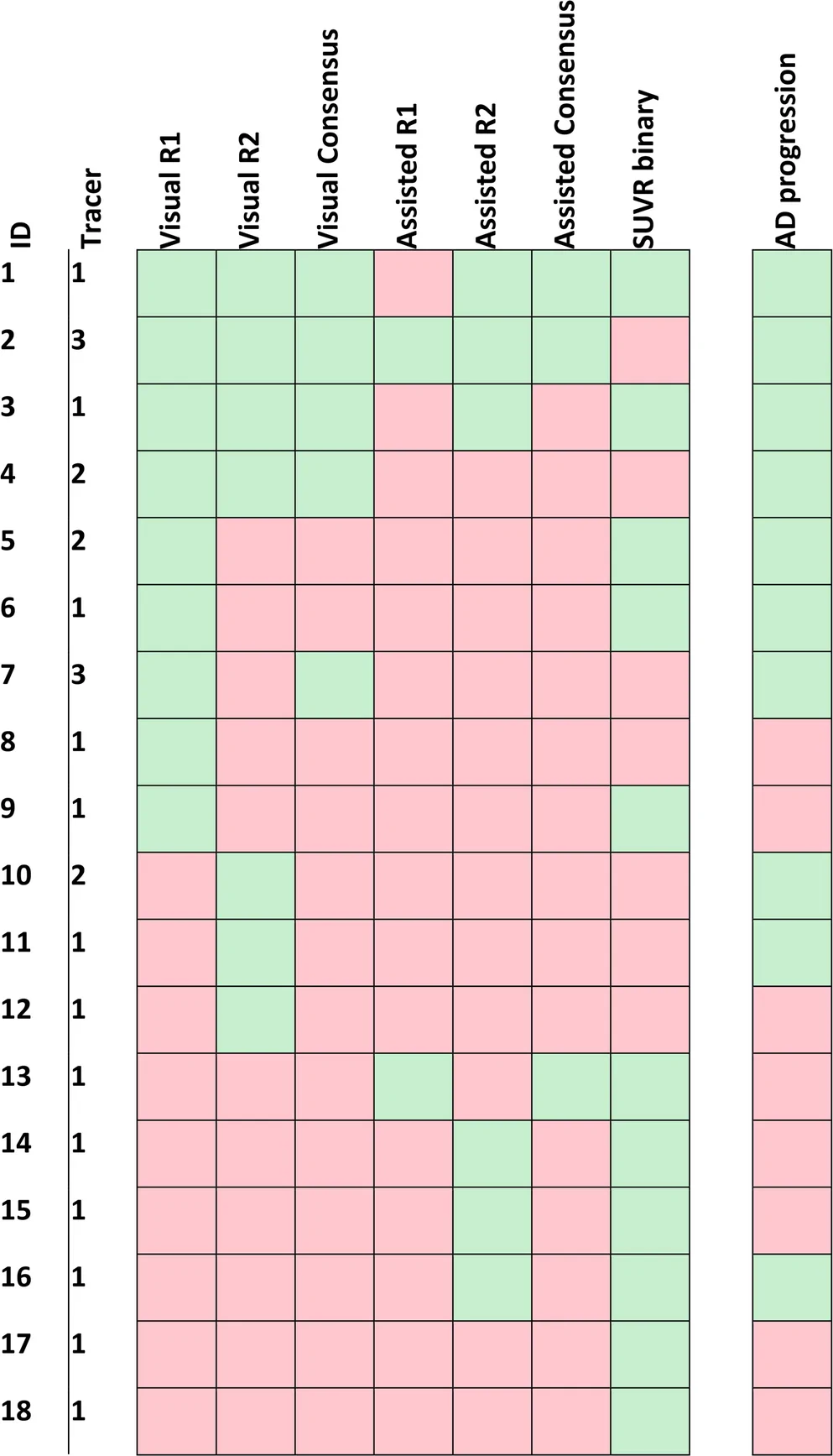
Discordant cases in amyloid PET classification. Heatmap of cases with discordant amyloid PET results, defined as disagreement between Reader 1 and Reader 2, or disagreement between visual assessment, consensus-assisted visual assessment, and SUVR-based classification. Green and red cells represent negative and positive binary classifications, respectively, illustrating the distribution and source of discordance across readers and assessment methods. 1 Florbetapir 2 Florbetaben 3 Flutemetamol

A secondary analysis focused on studies exhibiting inter-observer disagreement was conducted in order to characterise the quantitative and biological context of discordant interpretations (Fig. 7).

Of the total cohort of 145 amyloid PET studies, 18 cases (12.4%) showed disagreement between readers across at least one interpretative criterion. These cases were therefore classified as discordant and analysed separately to explore potential underlying patterns associated with reader variability.

In this secondary analysis of 18 discordant cases, conventional visual interpretation showed higher accuracy than assisted visual interpretation when clinical progression to AD was used as the reference. Reader 1 correctly classified 9 of 18 cases (50.0%) with conventional visual assessment, compared with 2 of 18 cases (11.1%) with assisted interpretation. Reader 2 correctly classified 7 of 18 cases (38.9%) with conventional visual reading, compared with 6 of 18 cases (33.3%) using the assisted method. Consensus-based interpretation followed the same pattern, with 5 of 18 cases (27.8%) correctly classified by visual consensus compared with 3 of 18 cases (16.7%) by assisted consensus. Thus, no reader-level or consensus-level advantage of assisted interpretation was observed in this clinically challenging subgroup; instead, conventional unaided visual assessment performed better overall. By comparison, SUVR binary classification correctly matched clinical progression in 11 of 18 discordant cases (61.1%). To further characterize the discordant cases, all 18 examinations were re-evaluated by FG (reader 2). Thirteen cases corresponded to [^18^F]-florbetapir, three to [^18^F]-florbetaben and two to [^18^F]-flutemetamol. Among the florbetapir cases, discordance was mainly related to quantitative assessment, with cortical atrophy considered a contributing factor in four examinations. The remaining florbetapir cases were considered intrinsically difficult to interpret because of image quality or subtle/borderline tracer uptake. In the few discordant florbetaben and flutemetamol studies, no consistent pattern was identified, and individual factors such as mild cortical atrophy or atypical regional uptake appeared to contribute to the discrepant interpretation. Because of the small number of discordant cases, these findings should be interpreted descriptively.

## Discussion

This longitudinal, real-world study with up to 8 years of clinical follow-up evaluated the prognostic value and reproducibility of three amyloid PET interpretation strategies in patients with amnestic mild cognitive impairment. Our findings confirm that amyloid PET positivity is a strong, independent predictor of conversion from aMCI to AD dementia. Amyloid PET positivity was consistently associated with progression to AD regardless of the interpretation method. Although assisted visual interpretation yielded the highest hazard ratio, the differences between methods were modest and should be interpreted cautiously, particularly considering the exploratory findings observed in the discordant subgroup. Overall, all three approaches provided clinically meaningful prognostic information. These findings are consistent with previous multicentre studies demonstrating the high diagnostic and prognostic value of amyloid PET and the strong concordance between visual interpretation and quantitative assessment in routine clinical practice [5, 6, 20]. In this context, our data fit well with the 2025 updated Appropriate Use Criteria, which support the use of amyloid PET in carefully selected patients when demonstration of amyloid pathology is likely to refine diagnostic confidence, prognostic stratification and clinical management [4].

Inter-observer agreement was excellent for both structured visual (κ = 0.877) and assisted visual interpretation (κ = 0.904), accompanied by high intra-observer reliability (κ = 0.860 for Reader 1; κ = 0.892 for Reader 2). These findings are aligned with previous studies. For instance, Takenaka et al. reported a kappa value of 0.83 among three expert readers interpreting [^18^F]-flutemetamol PET [21]. Similarly, high reproducibility has been reported for [^18^F]-florbetapir PET, with an inter-reader κ of 0.83 among five readers [22], and for [^18^F]-florbetaben PET, with κ values ranging from 0.89 to 0.94 [23]. These data support that the agreement observed in our real-world cohort is consistent with the reproducibility previously described for each individual amyloid PET radiotracer.

The excellent agreement observed for both readers suggests that standardized visual interpretation provides robust reproducibility, whereas quantitative tools may further support interpretation in selected situations. Although z-score overlays and SUVR thresholds offer modest improvements in prognostic performance and inter-reader agreement, these gains are relatively limited. Expert readers achieved excellent reproducibility using visual interpretation alone, suggesting that quantitative tools are most beneficial for improving standardisation and performance among less experienced readers, possibly in the evaluation of borderline cases.

This perspective is reinforced by Fleisher et al. who demonstrated that [^18^F]-Florbetapir PET, when interpreted visually by trained readers, accurately detects β-amyloid pathology in patients with mild cognitive impairment or AD dementia, with high sensitivity and specificity [24]. Similarly, Clark et al. (2011) demonstrated strong correlations between [^18^F]-Florbetapir PET signal and both the presence and density of β-amyloid plaques, supporting the reliability of expert visual interpretation [6].

A particularly informative aspect of our study was the analysis of discordant cases. In our cohort, 18/145 scans (12.4%) showed disagreement across at least one reader-level interpretative condition, a proportion that is broadly consistent with the high but not perfect agreement reported in amyloid PET studies. In multicentre datasets, binary visual interpretation has typically shown agreement rates around 90% with kappa values in the almost-perfect range, while disagreement tends to cluster in scans with low or borderline amyloid burden [25].

In this discordant subgroup, conventional visual assessment outperformed assisted interpretation for both readers when clinical progression to AD dementia was used as the pragmatic reference standard. This suggests that, although adjunctive tools can improve reproducibility at the cohort level, their effect is not necessarily uniform in clinically ambiguous cases. Prior studies have shown that semiquantitative support may improve inter-reader agreement, particularly in mixed-experience settings, but may offer less incremental value for readers who already perform at a high level or in scans that remain biologically borderline [26].

Because of the limited number of discordant cases (*n* = 18), this subgroup analysis was considered exploratory and descriptive; therefore, no formal statistical comparisons between interpretation methods were performed. A detailed review of the discordant examinations showed that disagreement was not random but concentrated in technically or biologically challenging studies. Most discordant cases involved [^18^F]-florbetapir, where quantitative discrepancies were frequently associated with cortical atrophy, whereas other cases reflected reduced image quality, borderline uptake or atypical regional tracer distribution. These findings suggest that discordant interpretations are more likely to arise from image characteristics than from intrinsic limitations of either visual or quantitative assessment, reinforcing the complementary role of expert visual interpretation and quantitative tools in difficult cases. At first glance, these findings may appear inconsistent with the higher hazard ratio observed for assisted visual interpretation in the Cox analysis. However, the discordant subgroup represented only a small exploratory subset of particularly challenging examinations and was not intended to compare the overall diagnostic performance of the different approaches. Taken together, our results suggest that quantitative tools may improve prognostic stratification at the population level while offering limited additional value in the small proportion of technically or biologically challenging cases encountered in routine clinical practice. Quantitative or visually assisted tools should not be viewed as universally superior replacements for expert visual interpretation, but rather as complementary aids whose value may depend on case complexity, tracer pattern and reader expertise. This perspective is especially relevant in real-world memory clinic populations, where a small subset of scans will remain difficult despite otherwise excellent overall agreement [27].

The increasing incorporation of quantitative tools into amyloid PET interpretation also has implications for the future integration of artificial intelligence (AI). Several machine learning algorithms have shown promising performance for automated amyloid PET classification [28]. However, as with current quantitative methods, these approaches should be regarded as complementary decision-support tools rather than substitutes for expert visual interpretation. Our findings reinforce the importance of maintaining expert clinical oversight, particularly in technically challenging or borderline cases where image interpretation remains nuanced.

The findings observed in this study have direct implications for clinical decision-making as amyloid PET becomes increasingly central to anti-amyloid treatment strategies. Although quantitative tools may improve reproducibility and support longitudinal monitoring, expert visual interpretation remains essential in routine clinical practice. At the same time, there is a growing trend to include Centiloid values in clinical reports because of their independence of radiotracer used and the added value for treatment selection and therapeutic monitoring. Importantly, the Summary of Product Characteristics of the three radiotracers explicitly recommends using quantitative analysis as complement to visual assessment, highlighting its role in ensuring consistent and reliable interpretation. Our findings are consistent with this framework, advocating for the integration of standardised quantitative measures, whether global SUVR-based or Centiloid-scaled, into diagnostic workflows to strengthen diagnostic confidence and optimise real-world clinical decision-making.

A key limitation of this study is the absence of post-mortem neuropathological confirmation. As a result, clinical progression to AD dementia was used as the reference standard for evaluating amyloid PET positivity. Although this approach is commonly used in longitudinal observational studies, it introduces potential bias related to clinical heterogeneity and diagnostic uncertainty. Similar methodologies have been used in prior research; Mathotaarachchi et al. employed clinical progression as a surrogate gold standard in the absence of autopsy confirmation, acknowledging its inherent limitations [29]. Although clinical conversion represents a pragmatic real-world outcome, its use highlights the need for cautious interpretation and reinforces the importance of incorporating complementary biomarkers to improve diagnostic accuracy. Additional limitations include that quantitative analyses were performed using tracer-specific SUVR thresholds rather than Centiloid values. At the time the study was conducted, Centiloid conversion had not yet been routinely implemented in our clinical workflow. Although tracer-specific thresholds were validated for each radiotracer, future studies should incorporate Centiloid harmonization to facilitate quantitative comparisons across different amyloid tracers. In addition, all PET examinations were reconstructed using the same institutional reconstruction protocol regardless of radiotracer. Although this ensured methodological consistency in this retrospective real-world cohort, differences in tracer characteristics and native spatial resolution may have influenced quantitative measurements and should be considered when interpreting the results.

## Conclusion

This study shows that amyloid PET positivity is a robust predictor of progression from amnestic MCI to Alzheimer’s disease dementia regardless of interpretation strategy. Although assisted visual and quantitative approaches may provide complementary prognostic information, expert visual interpretation showed excellent reproducibility and remained clinically reliable in this real-world cohort. These findings support the complementary integration of quantitative tools into routine clinical practice rather than their use as replacements for expert visual assessment.

## Data Availability

The datasets generated during and/or analysed during the current study are available from the corresponding author on reasonable request.

## Declarations

### Clinical trial number

Not applicable. It is not a clinical trial.
